# ELECTRIcity: An Efficient Transformer for Non-Intrusive Load Monitoring

**DOI:** 10.3390/s22082926

**Published:** 2022-04-11

**Authors:** Stavros Sykiotis, Maria Kaselimi, Anastasios Doulamis, Nikolaos Doulamis

**Affiliations:** School of Rural, Surveying and Geoinformatics Engineering, National Technical University of Athens, 15772 Athens, Greece; mkaselimi@mai.ntua.gr (M.K.); adoulam@cs.ntua.gr (A.D.); ndoulam@cs.ntua.gr (N.D.)

**Keywords:** NILM, non-intrusive load monitoring, transformers, energy disaggregation, imbalanced data, deep learning

## Abstract

Non-Intrusive Load Monitoring (NILM) describes the process of inferring the consumption pattern of appliances by only having access to the aggregated household signal. Sequence-to-sequence deep learning models have been firmly established as state-of-the-art approaches for NILM, in an attempt to identify the pattern of the appliance power consumption signal into the aggregated power signal. Exceeding the limitations of recurrent models that have been widely used in sequential modeling, this paper proposes a transformer-based architecture for NILM. Our approach, called ELECTRIcity, utilizes transformer layers to accurately estimate the power signal of domestic appliances by relying entirely on attention mechanisms to extract global dependencies between the aggregate and the domestic appliance signals. Another additive value of the proposed model is that ELECTRIcity works with minimal dataset pre-processing and without requiring data balancing. Furthermore, ELECTRIcity introduces an efficient training routine compared to other traditional transformer-based architectures. According to this routine, ELECTRIcity splits model training into unsupervised pre-training and downstream task fine-tuning, which yields performance increases in both predictive accuracy and training time decrease. Experimental results indicate ELECTRIcity’s superiority compared to several state-of-the-art methods.

## 1. Introduction

Non-Intrusive Load Monitoring (NILM), or energy disaggregation, is an efficient and cost-effective framework to reduce energy consumption [1]. Energy (or Electricity) disaggregation algorithms aim to infer the consumption pattern of domestic appliances by only analyzing the aggregated household consumption signal. This process can be viewed as the decomposition of the aggregate power signal of a household into its additive sub-components, i.e., power signals of each domestic appliance. Various NILM approaches have been proposed in the literature. Some of the most successful exploit deep learning structures, such as recurrent [2] or convolutional neural networks (CNN) [3], to extract information about individual appliance consumption. Even though these techniques have good performance in energy disaggregation tasks, there are some limitations and challenges. **Challenge 1**: These algorithms are easy to be trapped in the assumption that adjacent events in a sequence are dependent, while, as long as time passes, the interactions between remote events are faded. **Challenge 2:** Long Short-term Memory (LSTM) [1] models have a memory mechanism, that decides the worth-remembering information from the useless one, every time a new state is entered in the sequence. Thus, local dependencies are more powerful than global ones, and old -or infrequent- events are faded in case they do not appear regularly. Data balancing is a necessary prerequisite in these approaches, to maintain important information. **Challenge 3:** Temporal Convolutional Neural Network (CNN) architectures capture long-range temporal dependencies in time series, with the necessary adaptations that include residual connections and dilated convolutions, but require significant model depth to catch long-range dependencies.

In this study, we introduce ELECTRicity, a transformer-based framework for solving the NILM problem. transformers do not sequentially process data. Instead, they process the entire sequence of data, understand the significance of each part of the input sequence and assign importance weights accordingly, using attention mechanisms, to learn global dependencies in the sequence. Even though transformer architectures seem suitable for NILM challenges, their applicability is limited [4] due to lack of efficiency and computational complexity issues. To fully exploit the capabilities of the transformer-based architecture, ELECTRicity consists of two parts: ***(i) the pre-training process***, which is an unsupervised pre-training process that requires only the aggregated power signal as input, ***(ii) the training process***, in which the pre-trained transformer model is fine-tuned in a supervised way to predict the electrical consumption of a specific domestic appliance. During the pre-training step, our model consists of a transformer-based **generator** and a **discriminator**, that cooperate to increase model performance, while using few computational resources. This process lead to a novel, efficient NILM framework, that has the comparative advantages summarized below:

- **ELECTRIcity is capable of learning long-range temporal dependencies.** In seq2seq models, learning temporal dependencies is a demanding task, and often the model forgets the first part, once it completes processing the whole sequence input. ELECTRIcity utilizes attention mechanisms and identifies complex dependencies between input sequence elements regardless of their position.
- **ELECTRIcity can handle imbalanced datasets.** Our work demonstrates that combining the unsupervised pre-training process with downstream task fine-tuning, offers a practical solution for NILM, and handles successfully imbalanced datasets. This is a comparative advantage against the existing state-of-the-art NILM works which, in most cases, require data balancing to achieve good performance.
- **ELECTRIcity is an efficient and fast transformer.** ELECTRIcity introduces a computationally efficient unsupervised pre-training process through the combined use of a generator and a discriminator. This leads to a significant training time decrease without affecting model performance compared to traditional transformer architectures.

### Related Work

Deep learning has achieved great success in domains such as computer vision and natural language processing (NLP) [5]. Since 2015, deep neural networks (DNN) have transversed into NILM and the number of the proposed DNN approaches has increased rapidly [6].

Recurrent neural networks (RNN), LSTM, bidirectional LSTM (BiLSTM), and gated recurrent unit (GRU) networks have been firmly established as state-of-the-art approaches in NILM [7]. These techniques take advantage of recurrent mechanisms to identify temporal patterns in power consumption sequences. Recurrent layers utilize feedback connections to capture temporal information in ‘memory’ and are well suited to sequential power signal data and energy disaggregation tasks. However, RNN lacks the ability to learn long-range temporal dependencies due to the vanishing gradient problem, as the loss function decays exponentially with time [8].

LSTMs rely on memory cells that employ forget, input, and output gates to memorize long-term temporal dependencies [2]. Even though LSTMs are successful in several time-series-related tasks, their elaborate gating mechanism may result in increased model complexity. At the same time, computational efficiency is a crucial issue for recurrence-based models and alternative architectures, such as GRU networks, have been developed to alleviate this limitation. These have been widely proposed in NILM [9].

CNN-based architectures have made great progress towards capturing long-range temporal dependencies in time series [10], but require significant model depth to expand their receptive field. Various works have proposed CNN-based solutions that leverage emerging advancements like, for instance, causal or temporal 1D-CNN to address NILM-related challenges [3]. These networks combine causal, dilated convolutions and other model modification techniques, such as residual connections or weights normalization to limit computational complexity without affecting the model’s performance. Alternative approaches suggest hybrid CNN-RNN architectures, that benefit from the advantages of both convolutional and recurrent layers. Representative examples of how these hybrid structures can be applied to NILM are [11,12].

Sequence-to-sequence (seq2seq) models have been widely used for energy disaggregation [7]. These models are particularly successful at machine translation [13], where word sequences are translated from one language to another. By analogy, in the energy disaggregation field, the aggregated sequence is translated through a seq2seq model to the power consumption of a specific domestic appliance. Denoising autoencoders are commonly considered the current state-of-the-art deep learning method for NILM [6,14]. Apart from seq2seq models, sequence-to-point (seq2point) and sequence-to-subsequence (seq2subseq) methods have also been utilized.

Most of the aforementioned studies deploy a pre-processing strategy to handle data balancing properly. In a NILM framework, the time interval between an appliance being switched on and off is referred to as an activation [7]. Domestic appliances, depending on their household use, may showcase from zero to several activations daily. Usually, the appliance run-time is considerably shorter compared to the time it is switched off, which leads to skewed datasets with sparse appliance activations.

Transformers [15] have rapidly emerged across a wide variety of sequence modeling tasks [16,17,18], due to their ability to arbitrarily and instantly access information across time, as well as their superior scaling properties compared to recurrent architectures. The main advantage of transformers stems from the fact that they, in contrast to the aforementioned architectures, process a sequence in parallel in an order-invariant way. Techniques such as positional embeddings and attention masking are an integral part of transformer-based methodologies [19,20]. Original transformers do not rely on past hidden states to capture dependencies. On the contrary, they process a sequence as a whole, mitigating the risk to lose -or ‘forget’- past information. As a consequence, transformers do not suffer from long-range dependency issues, which is the main controversy in RNN. Even though transformer architectures seem suitable for NILM challenges, their applicability is limited [4] due to efficiency and computational complexity issues.

## 2. Background

### 2.1. NILM Problem Formulation

Let *M* be the number of household appliances and *i* be the index referring to the *i*-th appliance (i=1,…,M) [21]. The aggregate power consumption *x* at a given time *t* is the sum of the power consumption of the individual appliances *M*, denoted by $y_{i}$∀i=1,…,M. Thus, in a NILM framework [22], the total power consumption *x* at a given time *t* is:

(1) $$x\left(t\right)=\sum_{i=1}^{M}y_{i}\left(t\right)+\epsilon_{noise}\left(t\right)$$

where $\epsilon_{noise}$ describes a noise term. Our goal is to solve the inverse problem and estimate the appliance consumption patterns $y_{i}$, given the aggregate power signal *x*. Therefore, NILM is formulated as a blind-source separation problem that is highly undetermined, since there are infinite combinations of $y_{i}$ that reconstruct *x*.

NILM presents several significant challenges that need to be overcome. The power signal exhibits severe non-linearity, since the temporal periodicity of the individual appliance activation depends on contextual characteristics [1], i.e., geographic and socioeconomic parameters or even residents’ habits. This leads to diverse energy consumption patterns in households. Therefore, it is challenging to implement models with good generalization ability that achieve high performance when tested on unseen houses. Other notable challenges include long-range temporal dependencies in appliance activations, as well as dataset imbalance. Many appliances may not be turned on every day, and operate for a small period of time, resulting in their activation function being dominated by zeros.

### 2.2. Transformer Model Fundamentals

**Figure 1:**
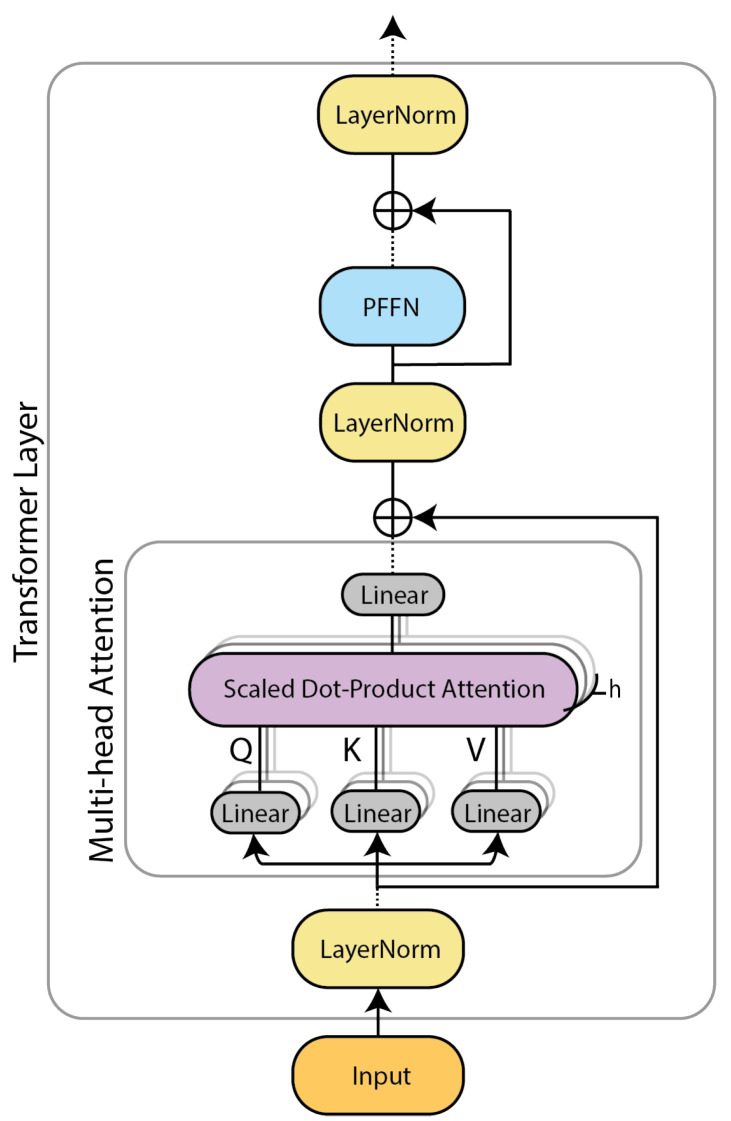
Overview of a transformer layer [15]. Dashed lines indicate Dropout regularization [23].

The transformer model [15] consists of two major components: a multi-head attention (MHA) module and a position-wise feed-forward network (PFFN). An overview of the transformer layer is depicted in Figure 1. The input signal is first normalized and fed to the multi-head attention layer, which calculates the attention scores (see Section 2.2.1). Then, the attention scores are normalized and passed on to a position-wise feed-forward layer (see Section 2.2.2). Residual connections and dropout regularization [23] are introduced to increase the stability of the model. In the following subsections, we shall introduce the two key components (MHA and PFFN) of a transformer layer.

#### 2.2.1. Multi-Head Attention Mechanism

Transformers implement attention mechanism as a Query-Key-Value (QKV) model. Attention consists of a series of linear transformations that process input sequences in an order-invariant way and assign importance weights to each position in the sequence. Thus, single-head dot-product attention mechanism applies linear transformations to the input signal to form query (Q), key (K) and value (V) matrices. Let us denote the input signal as $x\in R^{d_{b}\times d_{l}}$, where $d_{b}$ is the batch size and $d_{l}$ the input length. The linear transformations can be formulated as matrices $W_{q}\in R^{d_{l}\times d_{q}}$, $W_{k}\in R^{d_{l}\times d_{k}}$ and $W_{v}\in R^{d_{l}\times d_{v}}$.

(2) $$Q=W_{q}^{T}x,\;\;K=W_{k}^{T}x,\;\;V=W_{v}^{T}x$$

To ease matrix computations, $W_{q},W_{k}$ and $W_{v}$ should have the same size $d_{k}=d_{q}=d_{v}$. Single-head dot product attention (denoted by *A*) is then a matrix multiplication of *Q*, *K* and *V* after a scaling and softmax operation.

(3) $$A\left(Q,K,V\right)=softmax\left(\frac{QK^{T}}{\sqrt{d_{k}}}\right)V$$

**Figure 2:**
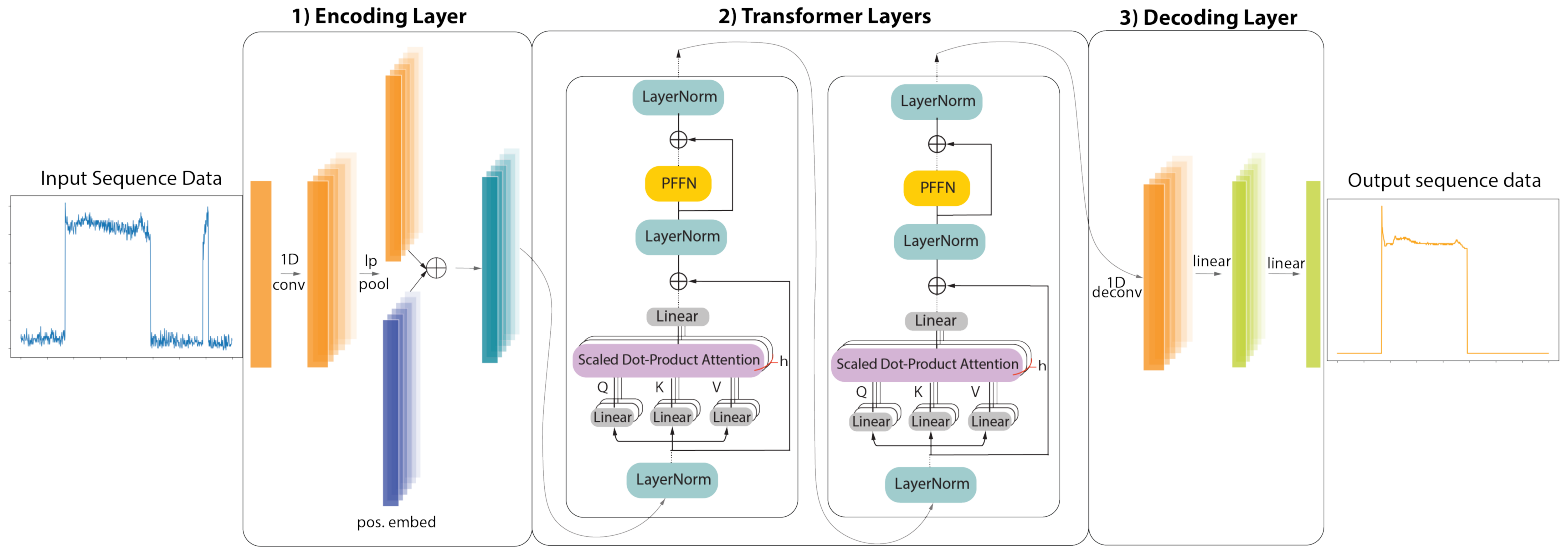
Proposed model architecture for ELECTRIcity’s generator and discriminator models. The architecture consists of 3 parts: (1) Encoding layer, which performs feature extraction from the input signal, (2) Transformer layers, which assign importance weights to the extracted features and (3) Decoding layer, which generates the desired output sequence.

The first term in Equation (3) can be viewed as the important weighting of values at all positions of the sequence. Therefore, attention can inherently understand which parts of the sequence are significant to predict the output and ignore parts that are not. This feature is particularly useful when dealing with imbalanced datasets since the respective weight for negative samples can automatically be set to a small value. Attention is an integral part of our proposed model architecture, which is illustrated in Figure 2.

Instead of simply applying a single attention function, transformers deploy a multi-head attention mechanism. MHA is calculated by extending the aforementioned single-head attention mechanism to h dimensions (multiple heads) by concatenating the single-head attention outputs, followed by a linear layer.

(4) $$MHA=Concat(A\left(Q_{i},K_{i},V_{i}\right))\;\forall i\in 1,\ldots h$$

In literature, multiple single-head attention techniques have been developed (additive attention [13], multiplicative attention [24], dot-product attention [15]). The latter is the most widely used variation.

#### 2.2.2. Position-Wise Feed-Forward Network

The normalized attention scores are passed on to a position-wise feed-forward layer (PFFN), which performs linear transformations with GELU activation function [25]. The linear transformations are applied to each position separately and identically, meaning that the transformations use the same parameters for all positions of a sequence and different parameters from layer to layer. Let us denote the attention sub-block output as *a* and the weight matrices and bias vectors of each linear transformation as $W_{1},b_{1}$ and $W_{2},b_{2}$ respectively. Then:

(5) $$PFFN\left(a\right)=GELU\left(0,aW_{1}+b_{1}\right)W_{2}+b_{2}$$

## 3. ELECTRIcity: An Efficient Transformer for NILM

ELECTRicity is an efficient model training routine for energy disaggregation. ELECTRicity splits model training into a pre-training (Section 3.1) and a training routine (Section 3.2). The pre-training step includes an unsupervised model trained with unlabeled data that uses only the aggregate signal and is applied for weight initialization to boost model performance. Here, during pre-training, we introduce the concept of generator and discriminator that is inspired by [26,27] to improve the efficiency of the proposed model. Then, the model is fine-tuned to handle the signal of an individual appliance [18] using the discriminator model.

### 3.1. ELECTRIcity’s Unsupervised Pre-Training Process

It is a common strategy in various transformer architectures to utilize a model pre-training procedure [4,18]. In such approaches, the model is pre-trained in an unsupervised way by replacing certain values from the input signal, and it is subsequently fine-tuned to solve any downstream task. Nevertheless, the loss function in such approaches [4,18] is calculated only considering the replaced positions, meaning that only a small fraction of the data is taken into account for model training. Even though it is an interesting technique, we argue that ignoring most output values is data inefficient and that a more effective strategy could lead to higher performance.

**Figure 3:**
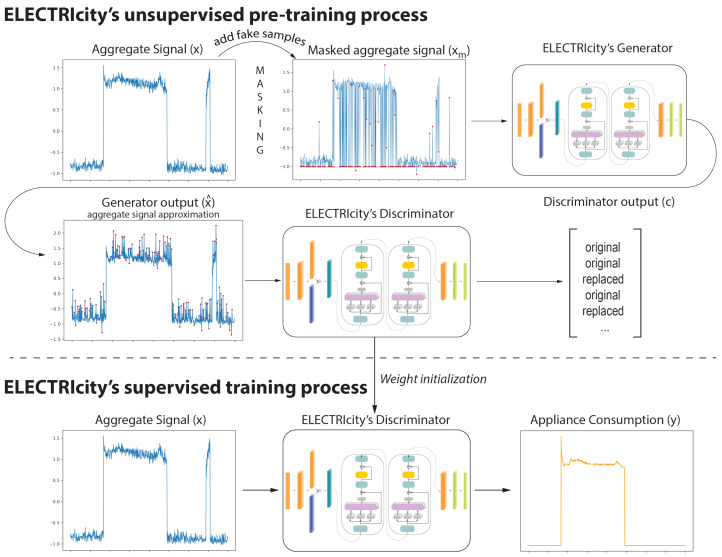
Overview of ELECTRIcity’s model training routine. Training is split into an unsupervised pre-training mechanism and a supervised process. During pre-training, the aggregate signal is masked at random positions with fake samples and the generator tries to reconstruct the original signal. The discriminator has to distinguish which positions of the generator output were fake (replaced) and which correspond to the original signal. During training, the generator is discarded and the discriminator is fine-tuned to predict the individual appliance consumption from the aggregate signal.

ELECTRicity’s efficient pre-training approach is illustrated in Figure 3. Contrary to the traditional transformer approaches described above, which use a single transformer model, ELECTRicity consists of two twin transformers, a generator, and a discriminator. In our approach, a fixed percentage of values in a given aggregate sequence $x\in R^{N}$ is masked/replaced to create a masked aggregate signal $x_{m}$. 80% of the masked samples are replaced with a predefined value (e.x. −1), 10% with a random value taken from a standard Gaussian distribution, and 10% with the original input value. The generator receives the masked aggregate signal and tries to predict the original signal values at the masking positions and reconstruct the original aggregate sequence. This procedure forces the model to understand the interdependencies of the aggregate sequence without relying on labeled data. The discriminator task is then to receive the generator estimation and understand which samples correspond to the aggregate signal and which were replaced.

To account for the data inefficiency of traditional masked pre-training mechanisms [4], the generator loss function is computed only on the masked portion of the signal, whereas the discriminator loss function utilizes the whole signal. The generator loss function consists of a combination of Mean Squared Error (MSE) and Kullback-Leibler Divergence ($D_{KL}$), while the discriminator loss function implements Binary Cross-Entropy (BCE) loss. To properly formulate the loss functions, let $x\in R^{N}$ be the aggregate signal and $\hat{x}\in R^{N}$ the generator output. Let further $m\in R^{N}$ be a binary mask with *M* masking positions and $x_{m}$ be the masked input signal. Finally, let *c* be the discriminators’ output. Then the pre-training loss functions $L_{gen}$ and $L_{disc}$ are:

(6) $$\begin{array}{cc} L_{gen} & =\frac{1}{T}\sum_{i=1}^{M}{\left(\hat{x_{i}}-x\right)}^{2}+D_{KL}\left(softmax\left(\frac{\hat{x}}{\tau }\right)∥softmax\left(\frac{x}{\tau }\right)\right)\\ L_{disc} & =-\frac{1}{N}\sum_{i=1}^{N}m_{i}log\left(p\left(c_{i}\right)\right)+\left(1-m_{i}\right)log\left(1-p\left(c_{i}\right)\right) \end{array}$$

where τ is a hyperparameter to control softmax temperature. From a dataflow perspective, the aggregate signal *x* is masked to produce $x_{m}$ that is used as input to the generator. The generator output $\hat{x}$ is passed on to the discriminator which predicts which values correspond to the original aggregate signal and which were replaced. That information is captured in vector *c*. This process can be summarized as:

(7) $$x\to x_{m}\to \mathrm{generator}\to \hat{x}\to \mathrm{discriminator}\to c$$

### 3.2. ELECTRIcity Supervised Training Process

On a high level, the pre-training process can be seen as a task-specific weight initialization technique to boost model performance. During training, the generator is discarded and the discriminator is re-trained to produce the appliance signature. Since, during training, the objective of the model changes, a different loss function is required that fits the energy disaggregation problem. The discriminator loss function is formulated in Equation (8).

(8) $$\begin{array}{cc} L(y,s) & =\frac{1}{N}\sum_{i=1}^{N}{\left({\hat{y}}_{i}-y_{i}\right)}^{2}\\ & +D_{KL}\left(softmax\left(\frac{\hat{y}}{\tau }\right)∥softmax\left(\frac{y}{\tau }\right)\right)\\ \; & +\frac{1}{N}\sum_{i=1}^{N}log\left(1+exp\left(\hat{s_{i}}s_{i}\right)\right)+\frac{\lambda }{N}\sum_{i\in O}\left|\hat{y_{i}}-y_{i}\right| \end{array}$$

where, λ is another hyperparameter that controls the impact of the absolute error from the set O of incorrectly predicted samples and timepoints when the status of the appliance is on. The loss function also considers the ground truth status of the appliance, as well as the on-off status *s* of the predicted consumption signal. During training, the dataflow is simpler. The aggregate signal *x* is used as input to the pre-trained discriminator, which outputs the individual appliance consumption signal *y*.

(9) x→discriminator→y

## 4. Experimental Results

We use three open-source datasets for results comparison, UK-DALE [28], REDD [29] and Refit [30]. All datasets include electricity measurements from multiple houses and provide both low-and high-frequency data. We focus on low-frequency data and will examine 4 appliance types: (1) Appliances with distant activations and very short activation period (Kettle, Microwave) (2) Appliances with frequent, recurring activations that do not have high power consumption peaks (fridge, fridge-freezer) (3) appliances with distant activations and long activation period (Washing Machine, Dishwasher) and (4) appliances with distant activations and low power consumption peak (TV). It should be noted that UK-DALE and Refit contain significantly more data than REDD and, therefore, more appliance activations.

The data was minimally processed to meet the requirements of Table 1. Aggregate and appliance signals were examined at $\frac{1}{6}$ Hz frequency and time gaps shorter than 3 min were forward-filled. No measures were taken to tackle class imbalance, as we would like to test to what extent the models can perform well in real life scenarios when the appliances are turned off most of the time. In the training set, the signals were split in windows of 480 samples (48 min) with a stride of 240 samples for UK-DALE and Refit and 120 samples for REDD. The models were tested on unseen data from a house not included in the training set without window stride. More specifically, in UK-DALE houses 1, 3, 4 and 5 were used for training and house 2 for testing. In REDD, house 1 was kept for model evaluation and houses 2, 3, 4, 5 and 6 were included in the training set, while in Refit houses 2, 3 and 16 were used for training and the models were tested on data coming from house 5.

To validate the performance of our methodology, we utilized several state-of-the-art models that are based on different technologies. More specifically, we adopted two recurrent approaches, GRU+ and LSTM+ [31], a convolutional seq2seq network [32,33] and a transformer-based solution [4]. The models were trained on a Google Colab server with an Nvidia Tesla P100 GPU.

In ELECTRIcity, both generator and discriminator followed the same architecture (Figure 2). Feature extraction was performed with a 1D-convolutional layer with kernel size 5 and a replicate padding of 2 on both sides. Feature extraction was followed by a squared average pooling layer with kernel size and stride 2. On the decoding side, a de-convolutional layer with kernel size 4, stride 2, and padding length 1 was implemented. Both models contain 2 transformer layers with 2 attention heads each and a hidden size $d_{k}$ of 64 for the generator and 64 for the discriminator. A Dropout probability of 10% has been adopted in all Dropout layers.

### 4.1. Performance Metrics

We recorded four widely used metrics to evaluate model performance. Mean Relative Error (MRE), Mean Absolute Error (MAE) and Mean Squared Error (MSE) (Equation (10)) were calculated using the ground truth and estimated appliance signature.

(10) $$MRE=\frac{1}{max(Y)}\sum_{i=1}^{N}|\hat{y_{i}}-y_{i}|,\;MAE=\frac{1}{N}\sum_{i=1}^{N}\left|\hat{y_{i}}-y_{i}\right|,\;MSE=\frac{1}{N}\sum_{i=1}^{N}{\left(\hat{y_{i}}-y_{i}\right)}^{2}$$

Accuracy and F1 score were also determined to assess if the model can properly address the class imbalance. The on-off status of the device is required and can be computed by comparing the appliance signature with the predefined requirements of Table 1. Accuracy is equal to the amount of correctly predicted time points over the sequence length, while F1-score is computed according to Equation (11), where TP stands for True Positives, FP for False positives and FN for false negatives.

(11) $$F1=\frac{TP}{TP+\frac{1}{2}\left(FP+FN\right)}$$

MRE, MAE and MSE indicate the model’s ability to correctly infer the individual appliance consumption levels, whereas F1-score indicates the model’s ability to adequately detect appliance activations in imbalanced data. In our study, F1-score is the most important metric, as it captures the model’s ability to identify appliance activations and minimize false positives.

### 4.2. Evaluation

**Figure 4:**
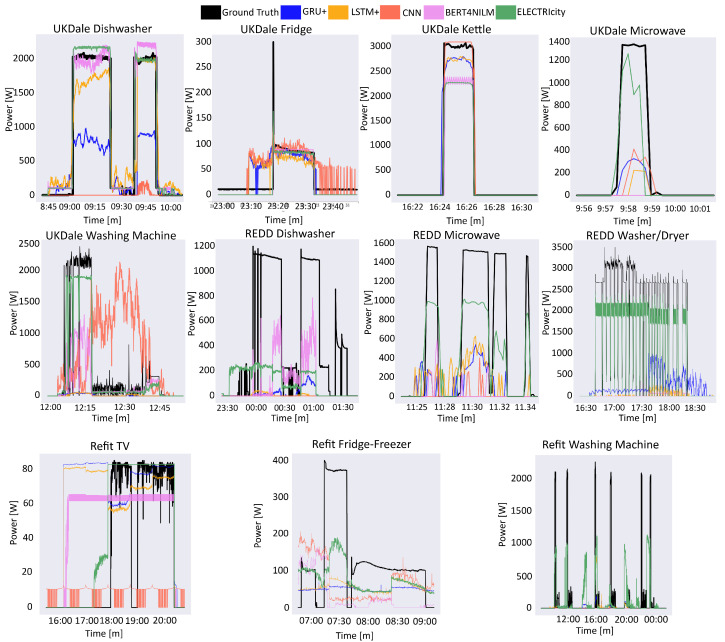
Comparison between ground truth appliance consumption signal and comparative model outputs for all examined appliances.

The experimental results for UK-DALE, REDD and Refit are presented in Table 2, Table 3 and Table 4 respectively, while Figure 4 illustrates prediction examples for each examined appliance. Across all datasets, ELECTRIcity outperforms the other models in most of the appliances.

Let us now consider the kettle and microwave appliances. For these appliances, ELECTRIcity showcases a performance increase in terms of F1-Score and, in some cases, a slightly lower MAE across both datasets (UK-DALE, REDD). This can be translated to a better model capability to detect activations, while not always reaching a precise consumption prediction, which can be explained by the high data sparsity due to the timespan of each activation. In these appliances, lighter models in terms of computational complexity (CNN, LSTM+, and GRU+) reach lower performance at a lower training time. It can be argued that there is a tradeoff between performance and computational complexity during training for these appliances. It should be mentioned that ELECTRIcity and the compared models (CNN, LSTM+, and GRU+) present similar computational demands during the testing phase, while ELECTRIcity has a higher performance. A different pre-training strategy, in the sense of using an alternative masking distribution, may lead to a further performance increase. In future work, we will evaluate such approaches to investigate the full capabilities of our model.

In the second case of experiments, we have examined the fridge in UK-Dale and fridge-freezer in Refit appliances. When disaggregating the fridge appliance, ELECTRIcity is outperforming most comparison models, but falls short to BERT4NILM [4]. The activations frequency for this appliance is unique, as it exhibits a periodicity that is usually not user-controlled. The fridge turns on when the inside temperature falls under a certain threshold, and turns off when that threshold is reached. Throughout a day, we can assume that the house temperature remains at a certain level, which in turn means that the periodicity of activations is constant and the appliance activates frequently. Therefore, a disaggregation model needs to capture the activation pattern very precisely to reach low regression errors and high classification performance. The masking procedure in the pre-training process of ELECTRIcity aims to model the noisy distributions in the aggregate signal, which is not suitable for constant recurring activations, as in the case of the fridge. On the contrary, the fridge-freezer appliance in the Refit dataset is different than the fridge, as it combines a periodic low-power activation with high consumption peaks stemming from the freezer cooling. Even though ELECTRIcity achieves the best MRE, it does not fully capture the activation pattern behavior, resulting in lower F1 score.

**Table 3:** Comparison of ELECTRIcity’s model performance to other techniques in the REDD dataset.

Device	Model	MRE	MAE	MSE	Acc.	F1	Training Time (min)
Washer	GRU+ [31]	0.028	35.83	87,742.33	0.985	0.576	2.94
	LSTM+ [31]	0.026	35.71	89,855.09	0.983	0.490	3.08
	CNN [32,33]	0.020	35.78	94,248.61	0.982	0.000	3.80
	BERT4NILM [4]	0.021	35.79	93,217.72	0.990	0.190	59.01
	ELECTRIcity	**0.016**	**23.07**	**44,615.35**	**0.998**	**0.903**	35.14
Microwave	GRU+ [31]	0.061	18.97	23,352.36	0.983	0.382	1.89
	LSTM+ [31]	0.060	18.91	24,016.75	0.983	0.336	1.53
	CNN [32,33]	0.056	18.07	24,653.65	0.987	0.336	2.22
	BERT4NILM [4]	**0.055**	16.97	22,761.11	0.989	0.474	27.72
	ELECTRIcity	0.057	**16.41**	**17,001.33**	**0.989**	**0.610**	16.49
Dishwasher	GRU+ [31]	0.049	24.91	22,065.08	0.962	0.341	2.76
	LSTM+ [31]	0.050	25.09	22,297.01	0.961	0.350	2.98
	CNN [32,33]	0.041	25.28	23,454.64	0.962	0.000	4.45
	BERT4NILM [4]	**0.038**	**19.67**	**15,488.62**	**0.974**	0.580	59.24
	ELECTRIcity	0.051	24.06	19,853.05	0.968	**0.601**	35.08

Next, we examine appliances with sparse, but longer duration activations (Washing Machine, Dishwasher), where ELECTRIcity showcases superior performance compared to the other models. For the washing machine, ELECTRIcity has better performance both in regression and in classification metrics. This performance increase is especially evident in the REDD dataset, where the F1 score is approximately 40% better than the second-best performing model. As for the dishwasher, its activation pattern is different than the washing machine, and contains more major fluctuations. ELECTRIcity produces a higher F1 score in both datasets, albeit with a lower MAE. This is due to the fact that the pre-training process of ELECTRIcity is suitable for modeling abnormal noisy distributions in the aggregate signal, which fits the activation profile of this appliance category. At the same time, ELECTRIcity requires 55% less training time than the second-best performing model (BERT4NILM) for the washing machine and 45% for the dishwasher, confirming the efficiency increase of our approach. We can therefore draw the conclusion ELECTRIcity is the most suitable model for disaggregation of the washing machine and the dishwasher.

In addition to the aforementioned appliances, we evaluate the disaggregation performance of an entertainment appliance (television). Entertainment appliances have particular disaggregation interest since they can be one of the main sources of energy saving for a domestic household. The television consumption pattern is different from the appliances examined so far, as the activations are distant and have a lower power consumption. Therefore, it is easier for the activations to be “lost” in the aggregate signal. However, our approach outperforms the other models both in regression and classification metrics, while requiring 75% less training time than the second-best performing model. This finding is very interesting and paves the way for evaluating ELECTRIcity on other entertainment appliances.

To summarize the above findings across all datasets, ELECTRIcity exhibits an average comparative performance increase of 9.03%, 5.38% and 23.59% in terms of MRE, MAE and MSE respectively, as well as an increase of 5.10% and 27.68% in terms of accuracy and F1-score to the second-best performing model [4], thus confirming the superiority of our approach.

**Table 4:** Comparison of ELECTRIcity’s model performance to other techniques in the Refit dataset.

Device	Model	MRE	MAE	MSE	Acc.	F1	Training Time (min)
Washer	GRU+ [31]	0.089	24.60	31,082.49	0.929	0.128	17.95
	LSTM+ [31]	0.098	25.76	32,958.09	0.920	0.130	22.86
	CNN [32,33]	0.096	23.58	29,383.90	0.924	0.248	28.62
	BERT4NILM [4]	**0.080**	**22.19**	27,420.48	**0.939**	0.188	813.25
	ELECTRIcity	0.089	23.67	**26,465.39**	0.936	**0.398**	217.32
TV	GRU+ [31]	0.619	38.36	2539.97	0.410	0.370	26.51
	LSTM+ [31]	0.657	39.35	2467.22	0.374	0.357	32.59
	CNN [32,33]	0.776	19.52	**980.39**	0.352	0.318	41.17
	BERT4NILM [4]	0.593	32.15	1769.43	0.452	0.381	1280.10
	ELECTRIcity	**0.278**	**19.29**	1375.13	**0.740**	**0.505**	316.10
Fridge-Freezer	GRU+ [31]	0.756	56.17	4773.60	0.552	0.710	17.95
	LSTM+ [31]	0.730	54.92	**4567.50**	0.551	0.710	22.86
	CNN [32,33]	0.686	58.15	5660.37	0.561	**0.713**	28.62
	BERT4NILM [4]	0.587	**50.16**	5437.78	**0.623**	0.674	813.25
	ELECTRIcity	**0.586**	51.08	5331.71	0.613	0.668	217.32

**Figure 5:**
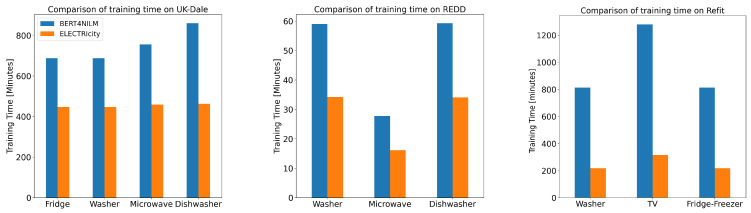
Training time comparison between transformer architectures on UK-Dale, REDD and Refit datasets. Averaging accross all datasets, our approach delivers a training time decrease of 50%.

Finally, we examine the performance advantages that the pre-training procedure yields in terms of training time between the two transformer-based models (ELECTRIcity and BERT4NILM). The total amount of training time per appliance can be seen in Figure 5. On average, ELECTRIcity required approximately 50% less training time than BERT4NILM using the same model size and hyperparameters. Overall, the introduction of a more efficient pre-training technique that is not limited to a percentage of the data leads to both performance and training time improvements, which makes ELECTRIcity a fast and efficient transformer architecture for energy disaggregation.

## 5. Conclusions

In this paper, we introduced ELECTRIcity, an efficient fast transformer-based architecture for energy disaggregation. ELECTRIcity outperforms state-of-the-art models in both examined datasets without requiring any data balancing. Averaging across all devices, ELECTRIcity achieves a performance boost across both datasets. The most significant increase can be showcased through the MSE and F1-score, where ELECTRIcity attains an average comparative increase of 23.59% and 27.68% respectively against the second-best performing model BERT4NILM [4]. At the same time, ELECTRIcity requires 50% less training time than BERT4NILM, making our approach superior in both performance and computational efficiency.

However, the performance evaluation of our approach has highlighted some limitations. In appliances with sparse and short activations, the increased training time of ELECTRIcity may not be always justified, compared to models with lower computational demands during the training phase. The disaggregation performance of the model, even though it outperforms the other comparative models, needs to be enhanced to solidify the preference toward ELECTRIcity, especially in cases where the pre-training masking procedure fails to model the noise distribution of the aggregate signal. At the same time, ELECTRIcity offers a great opportunity to improve the performance of NILM on appliances such as fridges or fridge-freezers, where the activation behavior is recurring at a similar consumption level. The results on entertainment appliances with small power consumption (and thus difficult to be disaggregated), such as the television, are very promising and open further research opportunities in that direction. Finally, we believe that our approach, which can work with minimal data pre-processing, is a big step towards the large-scale integration of NILM techniques in domestic households. With future improvements and optimizations, ELECTRIcity has the potential to enable efficient federated learning strategies, thereby increasing privacy for customers and significantly reducing data storage costs.

In future research, we will explore different pre-training strategies to assess their impact on different appliances and improve the capabilities of the model. Additionally, we aim to investigate the performance of our approach on less studied appliances related to entertainment, as information about power consumption for such appliances can lead to more environmentally aware consumption behaviors in domestic households. Finally, we aim to evaluate the potential of ELECTRIcity at different lower sampling rates, which could enable less intrusive metering approaches and lower storage costs for data generated by smart meters.

## Figures and Tables

**Table 1:** Pre-processing values for the REDD (upper), UK-DALE (middle) and Refit (lower) datasets.

Appliance	λ	Max. Limit	On Thres.	Min. on Duration [s]	Min. off Duration [s]
Washer	$10^{-3}$	500	20	1800	160
Microwave	1	1800	200	12	30
Dishwasher	1	1200	10	1800	1800
Fridge	$10^{-6}$	300	50	60	12
Washer	$10^{-2}$	2500	20	1800	160
Microwave	1	3000	200	12	30
Dishwasher	1	2500	10	1800	1800
Kettle	1	3100	2000	12	0
Washer	$10^{-2}$	2500	20	70	182
TV	1.5	80	10	14	0
Fridge-Freezer	$10^{-6}$	1700	5	70	14

**Table 2:** Comparison of ELECTRIcity’s model performance to other techniques in the UK-DALE dataset.

Device	Model	MRE	MAE	MSE	Acc.	F1	Training Time (min)
Kettle	GRU+ [31]	0.004	12.38	28,649.73	0.996	0.799	40.67
	LSTM+ [31]	0.004	11.78	28,428.10	0.997	0.800	34.70
	CNN [32,33]	**0.002**	**6.92**	16,730.81	0.998	0.889	51.87
	BERT4NILM [4]	0.003	9.80	16,291.56	0.998	0.912	697.87
	ELECTRIcity	0.003	9.26	**13,301.43**	**0.999**	**0.939**	294.37
Fridge	GRU+ [31]	0.797	31.47	1966.50	0.750	0.673	33.35
	LSTM+ [31]	0.813	32.36	2058.13	0.748	0.661	34.67
	CNN [32,33]	0.726	30.46	1797.54	0.718	0.686	44.43
	BERT4NILM [4]	**0.683**	**20.17**	**1087.36**	**0.859**	**0.831**	687.12
	ELECTRIcity	0.706	22.61	1213.61	0.843	0.810	428.79
Washer	GRU+ [31]	0.056	21.90	27,199.96	0.950	0.228	33.06
	LSTM+ [31]	0.055	23.42	32,729.26	0.950	0.221	34.14
	CNN [32,33]	0.023	15.41	25,223.21	0.984	0.518	44.46
	BERT4NIMLM [4]	0.012	4.09	4369.72	0.994	0.775	687.74
	ELECTRIcity	**0.011**	**3.65**	**2789.35**	**0.994**	**0.797**	462.95
Microwave	GRU+ [31]	0.015	7.16	8464.09	0.994	0.131	35.18
	LSTM+ [31]	0.014	6.60	7917.85	0.995	0.207	37.05
	CNN [32,33]	0.014	6.44	7899.43	0.995	0.193	47.32
	BERT4NILM [4]	0.014	6.53	8148.81	0.995	0.049	755.58
	ELECTRIcity	**0.013**	**6.28**	**7594.23**	**0.996**	**0.277**	518.93
Dishwasher	GRU+ [31]	0.035	28.60	43,181.30	0.975	0.722	44.31
	LSTM+ [31]	0.036	28.75	42,333.18	0.975	0.727	47.48
	CNN [32,33]	0.051	41.44	80,292.31	0.960	0.087	56.99
	BERT4NILM [4]	**0.026**	**14.11**	**14,676.17**	0.982	0.804	859.87
	ELECTRIcity	0.028	18.96	24,152.70	**0.984**	**0.818**	462.83

## Data Availability

Not applicable.
